# Evaluation of IHH, PTCH1, and SMO protein immunohistochemistry in the human mandibular condyle at fetal stages from 30 to 80 mm greatest length

**DOI:** 10.1002/ar.70059

**Published:** 2025-09-26

**Authors:** Filipe Santos da Silva, Carolina de Oliveira Gigek, Andreia Fabiana Do Vale Franco, Amanda Alves Ribeiro Massoni, José Ramon Mérida‐Velasco, Luís Otávio Carvalho de Moraes

**Affiliations:** ^1^ Department of Pathology Federal University of São Paulo São Paulo Brazil; ^2^ Department of Morphology and Genetics Federal University of São Paulo São Paulo Brazil; ^3^ Department of Anatomy and Embryology University Complutense of Madrid Madrid Spain

**Keywords:** fetal development, IHH protein, morphogenesis, PTCH1 protein, SMO protein, temporomandibular joint

## Abstract

This study evaluated the morphogenesis of the temporomandibular joint (TMJ) in human fetuses during the third month of gestation through the analysis of immunohistochemistry for the proteins Indian Hedgehog (IHH), Patched‐1 (PTCH1), and Smoothened (SMO). These proteins are critical components of the Hedgehog signaling pathway in the embryonic development. Together, they transduce essential intracellular signals for cartilage and bone development and regulate chondrocyte differentiation and growth as part of the synergistic molecular mechanisms that converge to form synovial joints, including the TMJ. A prospective observational study was conducted on six human fetuses at fetal stages ranging from 30 to 80 mm greatest length (estimated to range between 9 and 12 weeks gestational age). Hematoxylin–eosin staining was used for morphological analysis, and protein immunostaining was assessed through immunohistochemistry. The percentage of immunostaining was quantified using digital image analysis with ImageJ software. IHH immunostaining peaked at the 30 mm stage (4.63%), decreased at 60 mm (2.16%), increased at 70 mm (3.70%), and declined again at 80 mm (2.75%). PTCH1 showed the highest immunostaining at 30 mm (5.35%), with a progressive decrease to its lowest level at 80 mm (1.18%). SMO immunostaining was highest at 30 mm (4.07%), decreased at 60 mm (1.80%), and increased at 70 mm (2.63%) and 80 mm (3.52%). Strong correlations were found between IHH and PTCH1 (rho = 0.70) and between IHH and SMO (rho = 0.70), while PTCH1 and SMO showed a moderate correlation (rho = −0.30). These findings highlight the dynamic protein activity and their critical roles in TMJ morphogenesis.

## 1 INTRODUCTION

The temporomandibular joint (TMJ) is a synovial joint that, indirectly, holds greater importance in human development compared to any other joint in adults (Burch, 1970; Stocum & Roberts, 2018). It plays a fundamental role in essential activities for maintaining life, such as chewing, breathing, and communication (Roberts & Stocum, 2018).

The embryological development of the TMJ joint results from the interplay of environmental and genetic factors that contribute to forming this highly adaptable structure. Its movements are not only guided by bony coupling, ligaments, and musculature but also by dental occlusion, as both TMJs are connected by the mandibular bone and cannot move independently (Alomar et al., 2007).

The microstructure of the TMJ condyle cartilage consists of five histological layers, as proposed by de Ferraris and Muñoz (1999). Articular surface: single layer of flattened cells; mesenchymal layer: layer of rounded cells; chondroblastic layer: multiple chondrocyte cells surrounded by a sparse cartilaginous matrix; chondrocyte layer: chondrocytes embedded in a slightly basophilic matrix; and hypertrophic chondrocyte layer: apoptotic chondrocytes replaced by osteocytes. This deepest layer is the site of endochondral ossification and plays a key role in the cartilage's adaptability during mandibular development (de Ferraris & Muñoz, 1999).

Recently, a significant number of studies have covered a variety of topics on the TMJ, from embryogenesis and joint development to pathologies (Chen et al., 2017; Herrera‐Valencia et al., 2020; Idáñez‐Robles et al., 2023).

With regards to morphogenesis, from the several molecular regulatory pathways involved in the formation of the TMJ, the main ones include the Hedgehog signaling pathway (IHH), the Wnt/β‐catenin signaling pathway, the Bone Morphogenetic Proteins (BMP) signaling pathway, and the Transforming Growth Factor β (TGF‐β) signaling pathway. These pathways are crucial for signaling and regulating joint development and are vital for cell proliferation, differentiation, and migration (Lu et al., 2022; Niu et al., 2016; Shibukawa et al., 2007). Studies in mice have demonstrated that activation or inhibition of these pathways can result in TMJ malformations, highlighting their importance in proper joint formation (Mau et al., 2007; Shibukawa et al., 2007).

IHH is essential for regulating chondrocyte differentiation and the transition to hypertrophic cartilage. PTCH1 functions as a receptor in the Hedgehog signaling pathway, mediating responses to IHH. SMO, another critical component of this pathway, transduces intracellular signals essential for cartilage and bone development. Together, these proteins provide a comprehensive understanding of the molecular mechanisms involved in this critical stage of development (Ruiz i Altaba, 2011; St‐Jacques et al., 1999).

The IHH has been identified as one of the key molecular regulators in TMJ formation in mice (Shibukawa et al., 2007; Yang et al., 2016). Research conducted in rats revealed that the absence of IHH leads to deficient development of the condylar cartilage, as well as irregular patterns of gene expression and chondrocyte maturation. Additionally, there was an absence of articular disc and joint cavity formation (Shibukawa et al., 2007).

The macro‐ and microscopic anatomical components of the TMJ have been extensively studied. Our research group has played a significant role in elucidating these components, particularly during TMJ morphogenesis. However, there is a lack of information regarding the molecular mechanisms involved in its formation, especially in humans (Shibukawa et al., 2007).

Existing literature primarily focuses on studies of other joints, particularly those of the upper and lower limbs, which provide valuable insights into the pathways and signaling molecules involved in the formation of synovial joints (Koyama et al., 2007; Pacifici, 2006).

To date, there is a scarcity of studies addressing this molecular signaling in the biological process of human TMJ development. Much of the current knowledge about TMJ development originates from experimental models (Nickel et al., 2018). This gap in the literature underscores the importance of this and future studies utilizing human samples to better understand the normal anatomical aspects of the TMJ, potentially contributing to the comprehension of abnormal and pathological conditions that may affect this structure.

Descriptive studies in humans have reported that TMJ development can be divided into three phases. The first is the blastematic stage (weeks 7–8 of development), which corresponds to the initial organization of the condyle, articular disc, and capsule. During week 8, intramembranous ossification of the temporal squamous bone begins. The second stage is the cavitation stage (weeks 9–11 of development), during which the inferior joint cavity begins to form (week 9), and condylar chondrogenesis is initiated. By week 11, the superior joint cavity starts to organize. The third stage is the maturation stage (after week 12 of development) (Mérida‐Velasco et al., 1999). In this context, the purpose of this study was to track the expression of IHH, PTCH1, and SMO proteins in human specimens at 9–12 weeks of gestation, considering their specific roles in mandibular condyle development.

## 2 MATERIALS AND METHODS

### 2.1 Research ethics committee

The current study is approved by the Ethics Committee of the Federal University of São Paulo under the number: 70275823.7.0000.5505.

### 2.2 Fetuses selections

This study is a prospective observational study that used a case series to investigate the temporomandibular joint in six human fetuses between the 9th and 12th weeks of intrauterine life, a developmental window that encompasses the cavitation stage (weeks 9–11), when inferior and superior joint cavities form, and the beginning of the maturation stage (week 12), when the synovial membrane becomes functional and condylar cartilage undergoes critical differentiation. The samples were curated and maintained by the principal investigator at the Department of Descriptive and Topographic Anatomy, within the Department of Morphology and Genetics, Paulista School of Medicine, Federal University of São Paulo, and their use for research purposes was approved by the Institutional Research Ethics Committee (protocol no. 70275823.7.0000.5505).

The following table provides an overview of the fetuses used in the study, including gestational age, type of analysis performed, and the sectional plane adopted. Hematoxylin and eosin (HE) staining and immunohistochemical analyses were conducted on frontal sections of the fetuses' temporomandibular joints. Table 1 details the identification of the fetuses, their size in millimeters (mm), corresponding gestational age, and the type of analysis performed in each case.

**TABLE 1:** General data of the fetuses and experiments performed.

Identification	Size (mm)	Gestational age (weeks)	Sectional plane	Type of experiment			
				HE	IHH	PTCH1	SMO
Fetus 1	30	9th week	Frontal	✔			
Fetus 1	30	9th week	Frontal		✔	✔	✔
Fetus 2	40	9th week	Frontal	✔			
Fetus 2	40	9th week	Frontal		✔	✔	✔
Fetus 3	60	10th week	Frontal	✔			
Fetus 3	60	10th week	Frontal		✔	✔	✔
Fetus 4	70	11th week	Frontal	✔			
Fetus 5	70	11th week	Frontal		✔	✔	✔
Fetus 6	80	12th week	Frontal	✔			
Fetus 6	80	12th week	Frontal		✔	✔	✔

Abbreviations: HE, hematoxylin and eosin; IHH, Indian Hedgehog; PTCH1, Patched‐1; SMO, Smoothened.

The fetal ages, in weeks, were determined by measuring the distance between the external occipital protuberance and the coccyx—greatest length (GL) (O'Rahilly & Müller, 2010; O'Rahilly & Müller, 2001). A standard measuring tape with 0.5 cm intervals was used for this measurement. Each fetus was measured by the same observer at three distinct moments. The simple arithmetic average of the three measurements was considered for the estimation of fetal age.

### 2.3 Sample preparation for paraffin embedding

For macroscopic examination, the temporomandibular joint regions were dissected with the help of a D. F. Vasconcellos M900® surgical microscope, as follows: the skin and subcutaneous tissue were retracted, exposing the masseteric fascia, the masseter muscle, the lateral ligament, the articular capsule, the temporal fascia, and the temporal muscle. Through the middle fossa of the skull, the dura mater was retracted, the squamous part of the temporal bone was removed, the lateral pterygoid muscle was dissected, and the periosteum of the mandibular fossa roof was removed (Moraes et al., 2008; Takagi et al., 1989). Finally, the temporomandibular joint samples were stored in 2% buffered formalin.

The temporomandibular joints were decalcified in EDTA for 2–3 h and washed in running water for 10 min. They were then placed in buffered formaldehyde for 24 h for fixation. Subsequently, the following dehydration procedure was performed: 50% ethanol for 24 h, 70% ethanol twice, every 6 h, absolute ethanol twice, and finally, they were clarified in xylene for 2 h and embedded in paraffin (Moraes et al., 2015; Takagi et al., 2005).

### 2.4 Hematoxylin and eosin staining

The protocol was carried out as follows: the slides were immersed in xylene for 5 min (twice), followed by immersion in absolute ethanol for 2 min (twice), 95% ethanol for 2 min, and washed in running distilled water. They were then stained with hematoxylin for 2 min, washed in distilled water and running water, and treated with 50% ethanol for 2 min, 80% ethanol for 2 min, and 95% ethanol for 2 min. After staining with eosin for 1 min, the slides were treated again with 95% ethanol (twice) for 2 min each, followed by 100% ethanol (three times) for 2 min each. Finally, the slides were immersed in xylene for 5 min (twice) and mounted with coverslips and Permount.

### 2.5 Immunohistochemical protocol

Immunohistochemical reactions were performed for the antigen of the proteins IHH, PTCH1, and SMO.

The deparaffinization was carried out in an oven at 65°C for 40 min. The antigen retrieval of the samples was done in citrate buffer solution (0.3M, pH 6.0) in a pressure cooker at maximum power for 15 min. Then, the slides were treated to block endogenous peroxidase by applying 3% hydrogen peroxide for 15 min. Afterward, the samples were washed with running water and in PBS solution. The sections were incubated in a humid chamber at room temperature for 1 h with the following antibodies: IHH (polyclonal, dilution 1:100, Sigma‐Aldrich™, Burlington, MA), PTCH1 (polyclonal, dilution 1:400, Sigma‐Aldrich™, Burlington, MA), and SMO (polyclonal, dilution 1:50, Santa Cruz Biotechnology™, Santa Cruz, CA).

Signal amplification was performed using the Novolink™ (Polymer) kit, following the protocol adapted from the manufacturer's manual. The sections were incubated with Post Primary for 30 min, washed again with washing buffer, and incubated with Novolink Polymer for 30 min. After another wash with washing buffer, peroxidase activity was developed using DAB (Diaminobenzidine) for 5 min. The slides were washed with running water, counterstained with hematoxylin, and washed again with running water.

Finally, the slides were dehydrated through an ascending series of ethanol concentrations and cleared in three baths of xylene. The slides were then mounted with a coverslip using Entellan resin (Sigma™), analyzed, and digitally photographed under the Olympus BX60 microscope with the OPTHD™ software.

The positive controls were tissues recommended by the manufacturer. The negative controls underwent all the steps of the immunohistochemical reaction described above, except for the incubation with the primary antibody, which was replaced by a buffer solution.

### 2.6 Immunohistochemical analysis

The immunostaining was analyzed using ImageJ software. Images were captured with a light microscope (Olympus BX60) with the assistance of the Opticam Microscopy OPTHD software (Olympus Corporation, Tokyo, Japan) at 10× magnification, with fixed focus and field clarity in regions where the tissue was intact. The analysis was performed on the entire area of the mandibular condyle.

The software was calibrated before quantification, and the Colour Deconvolution method was applied (Landini et al., 2021). For the vectors, the H DAB option was selected, isolating the brown staining corresponding to the DAB marking. The threshold range was then defined to identify the areas of positive staining.

### 2.7 Statistical analysis

The statistical analysis was conducted using the Jamovi software version 2.3.24. For descriptive analyses, categorical variables were presented as frequency (%). To evaluate the correlation between the immunostaining of the proteins PTCH1, IHH, and SMO at different gestational ages, the Spearman correlation test was used to assess the strength of the correlation between these variables. The Spearman correlation test measures the strength of the correlation between two categorical variables, and the significance of this test indicates that the correlation was different from “0” (null correlation) in relation to the hypothesis test, where the null hypothesis is rho = 0 and the alternative hypothesis is rho ≠ 0. However, the significance does not show the strength of the correlation. Values close to 0 indicate weaker or no correlations. For the interpretation of the correlation coefficient, the following values were used: 0.90–1.00 = Very high correlation; 0.7–0.89 = High correlation; 0.5–0.69 = Moderate correlation; 0.3–0.49 = Low correlation; 0–0.29 = Insignificant correlation (Hinkle et al., 2003).

## 3 RESULTS

### 3.1 Morphological assessment by hematoxylin and eosin staining

**FIGURE 1:**
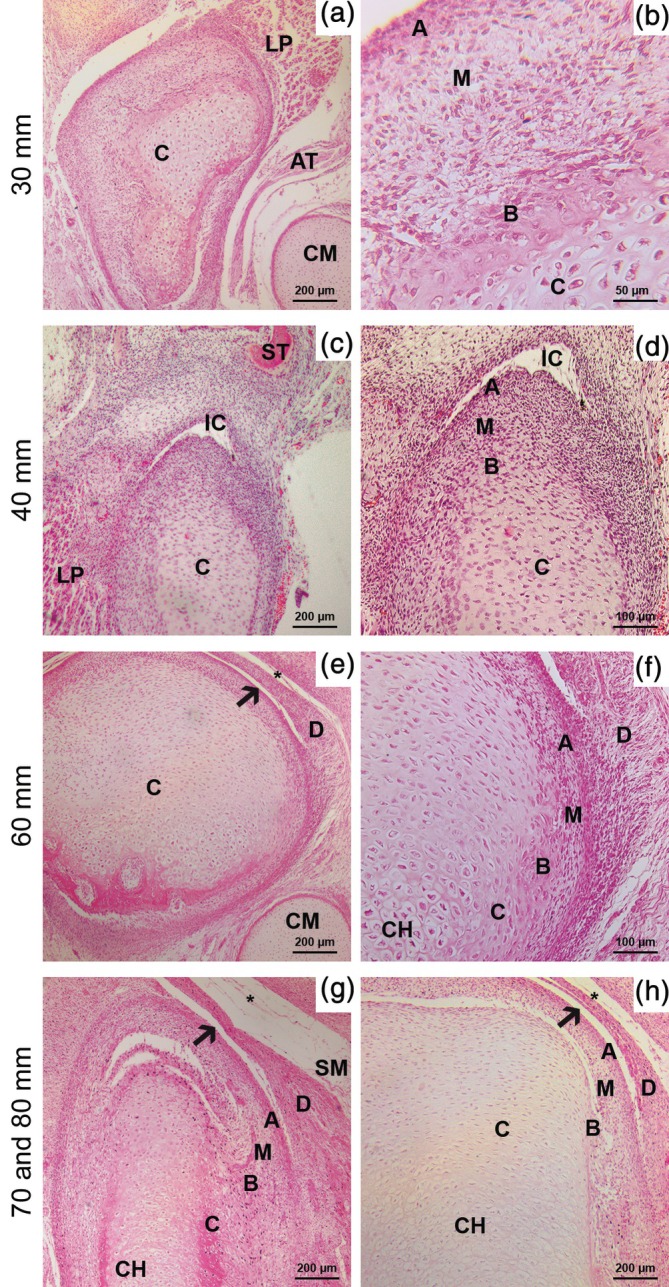
Histological analysis. Frontal sections stained with hematoxylin and eosin. (a, b) 30 mm GL (beginning of the 9th week); (c, d) 40 mm GL (end of the 9th week); (e, f) 60 mm GL (10 weeks of development); (g) 70 mm GL (11 weeks of development); (h) 80 mm GL (12 weeks of development). C, mandibular condyle; AT, auriculotemporal nerve; LP, lateral pterygoid muscle; CM, Meckel's cartilage; ST, squamous part of the temporal bone; IC, inferior articular cavity; D, articular disc; SM, synovial membrane; A, articular layer; M, mesenchymal layer; B, chondroblastic layer; C, chondrocyte layer; CH, hypertrophic chondrocyte layer; arrow: inferior articular cavity; asterisk: superior articular cavity.

At the fetal Stage 30 mm GL (Figure 1a,b), the condylar blastema, Meckel's cartilage, auriculotemporal nerve, and lateral pterygoid muscle are observed. The mandibular condyle begins to show zone differentiation, with the articular layer, mesenchymal layer, and a layer of polymorphic cells with scarce intercellular matrix (chondroblastic layer) becoming evident. Additionally, the chondrocyte layer starts to distinguish itself.

At the 40‐mm GL (Figure 1c,d), the mandibular condyle exhibits greater organization, with a clearer separation of the zones within the condylar cartilage. The superficial articular layer shows grouped cell bodies, while the mesenchymal layer contains small, rounded cells. The chondroblastic layer displays cells with intense mitotic activity and a notable increase in the chondrocyte layer, indicating cartilage growth. During this week, the initial formation of the inferior joint cavity is also evident.

At the 60‐mm GL (Figure 1e,f), both the inferior and superior joint cavities are observed, indicating significant progress in TMJ morphology and formation. During this period, in addition to the previously mentioned cartilage layers, a distinct layer of hypertrophic chondrocytes becomes apparent, aligning in columns perpendicular to the condylar articular surface.

At the 70–80 mm GL, the synovial membrane is observed (Figure 1g), and the hypertrophic chondrocyte layer increases significantly (Figure 1h). The mandibular condyle presents clear signs of maturation.

### 3.2 Immunohistochemical analysis

**FIGURE 2:**
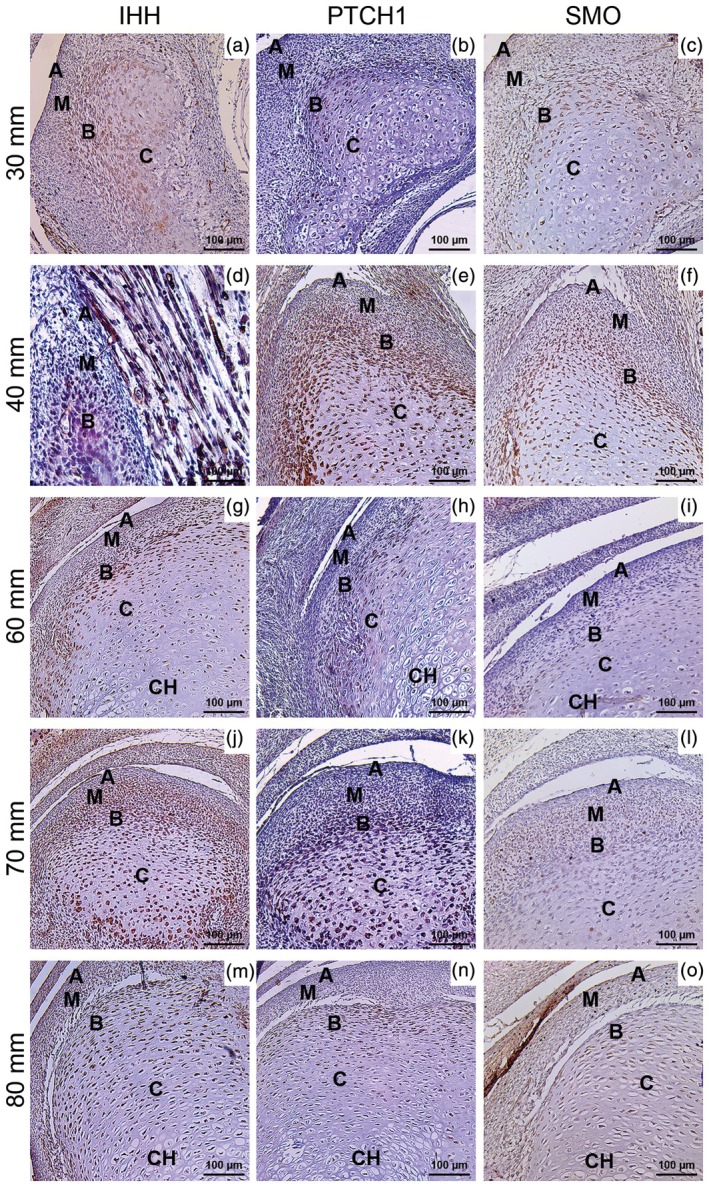
Immunohistochemical analysis for IHH, PTCH1, and SMO. (a–c) 30 mm GL (beginning of the 9th week); (d–f) 40 mm GL (end of the 9th week); (g–i) 60 mm GL (10 weeks of development); (j–l) 70 mm GL (11 weeks of development); (m–o) 80 mm GL (12 weeks of development). A, articular layer; M, mesenchymal layer; B, chondroblastic layer; C, chondrocyte layer; CH, hypertrophic chondrocyte layer.

Figure 2 shows the immunohistochemistry images for IHH, PTCH1, and SMO in the mandibular condyle of human fetuses at fetal stages from 30 to 80 mm greatest length.

IHH showed the highest staining values at the fetal Stage 30 mm GL (4.62%), with a progressive reduction until the stage of 60 mm (2.16%). From the stage of 70 mm to onward, there was an increase (3.70%), followed by a decrease at stage 80 mm (2.75%).

PTCH1 had the highest total staining at the fetal Stage 30 mm (5.35%) and showed a significant decrease in the following weeks, with the lowest value at stage 80 mm (1.18%).

SMO also demonstrated significant staining at the fetal stage of 30 mm (4.07%), with a reduction until the stage of 60 mm (1.80%). However, a new increase was observed at the stage of 70 mm (2.63%) and stage 80 mm (3.52%).

**FIGURE 3:**
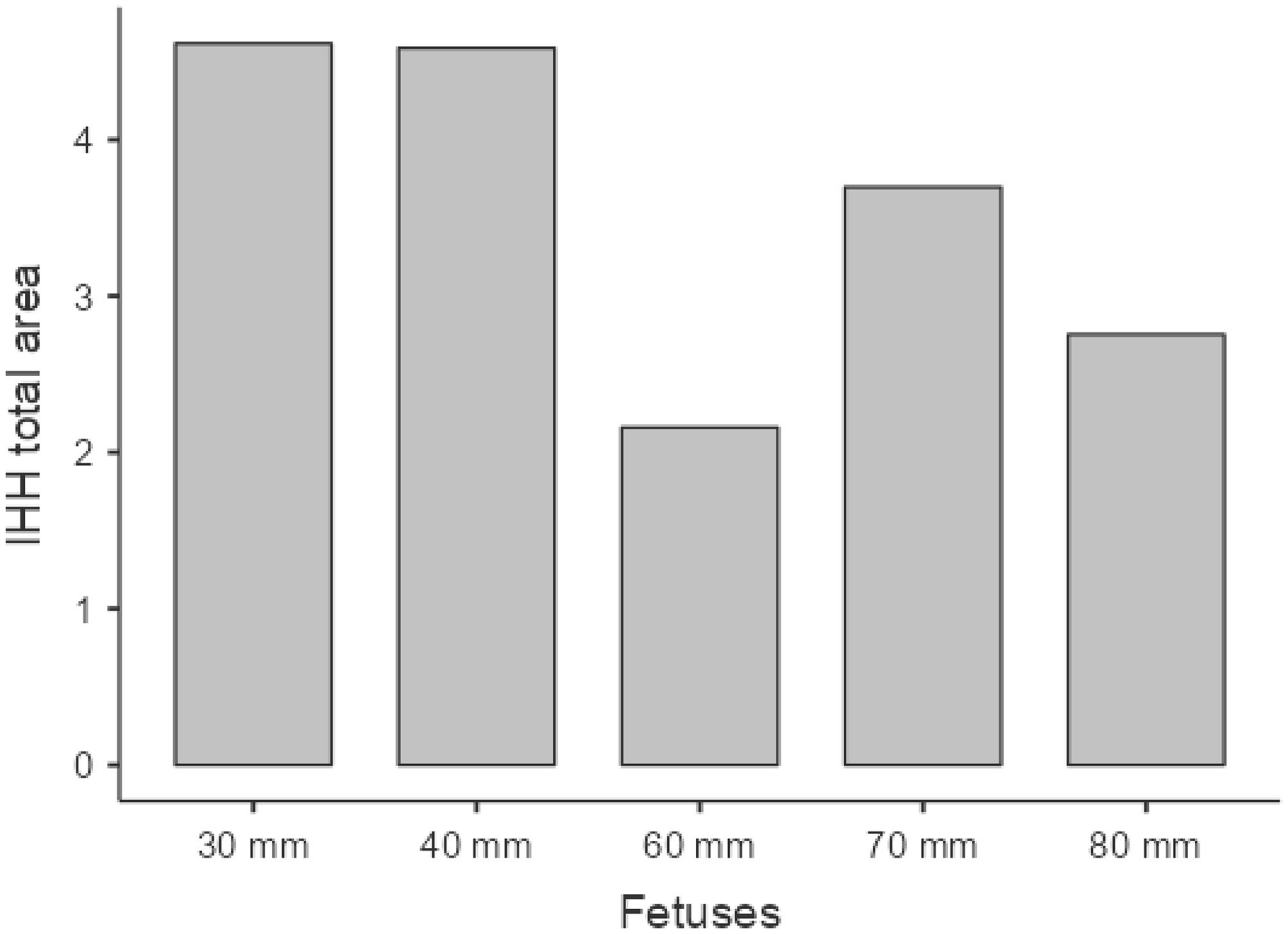
Percentage of IHH staining in the total area of the mandibular condyle.

**FIGURE 4:**
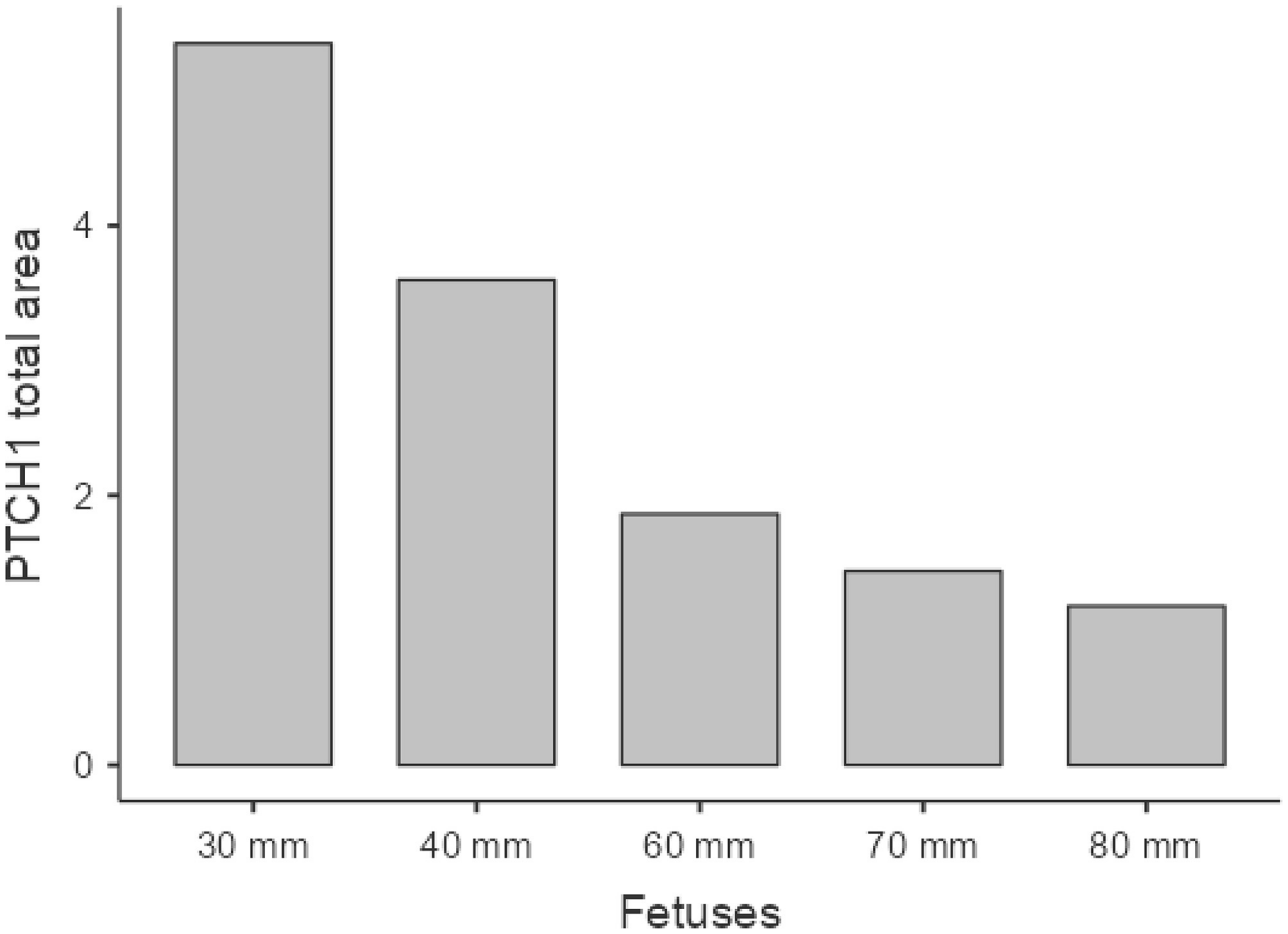
Percentage of PTCH1 staining in the total area of the mandibular condyle.

**FIGURE 5:**
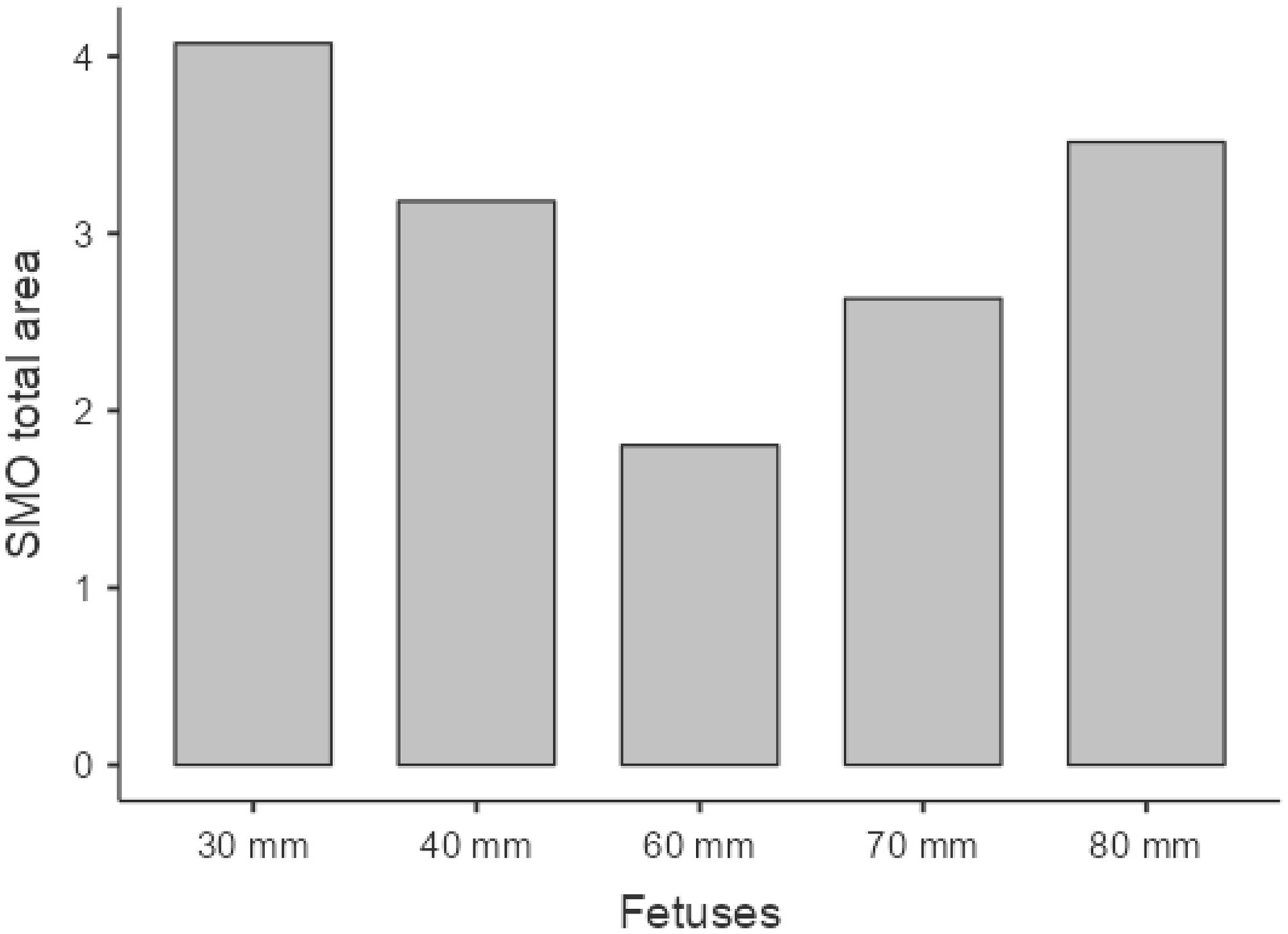
Percentage of SMO staining in the total area of the mandibular condyle.

The bar graphs shown in Figures 3, 4, 5 present the percentage of staining area for the proteins IHH, PTCH1, and SMO at fetal stages from 30 to 80 mm greatest length, with variations throughout the development of the mandibular condyle.

**FIGURE 6:**
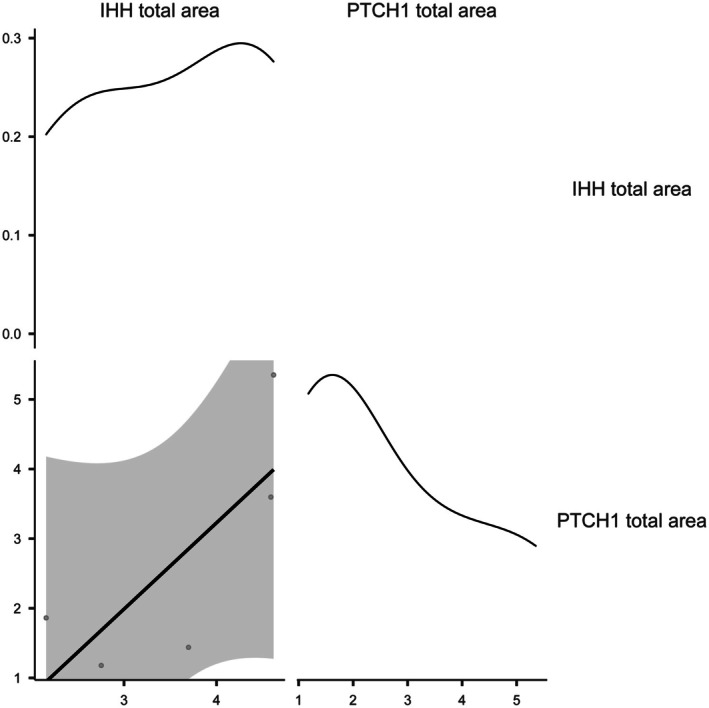
Correlation graph of IHH and PTCH1. Very high correlation: 0.90–1.00. High correlation: 0.7–0.89. Moderate correlation: 0.5–0.69. Low correlation: 0.3–0.49. Insignificant correlation: 0–0.29.

**FIGURE 7:**
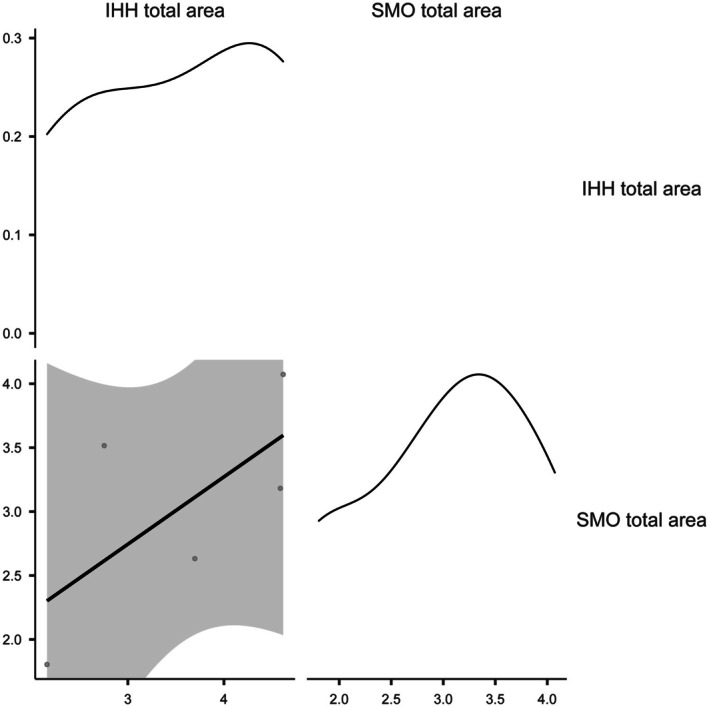
Correlation graph of IHH and SMO. Very high correlation: 0.90–1.00. High correlation: 0.7–0.89. Moderate correlation: 0.5–0.69. Low correlation: 0.3–0.49. Insignificant correlation: 0–0.29.

**FIGURE 8:**
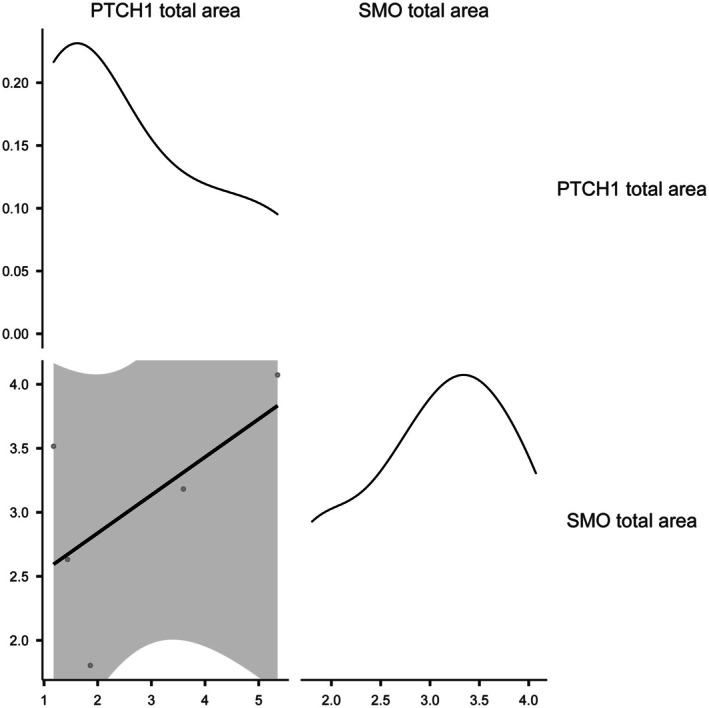
Correlation graph of PTCH1 and SMO. Very high correlation: 0.90–1.00. High correlation: 0.7–0.89. Moderate correlation: 0.5–0.69. Low correlation: 0.3–0.49. Insignificant correlation: 0–0.29.

The correlation analysis between the variables: staining area of IHH, PTCH1, and SMO is presented in a correlation matrix in Table 2. The percentages of the total staining area for PTCH1 and IHH proteins showed a high correlation (Spearman's rho = 0.70), as well as the total area for IHH and SMO proteins (Spearman's rho = 0.70). However, the quantification of the total area for PTCH1 and SMO proteins showed a moderate correlation (Spearman's rho = 0.30). The correlation graphs and densities are presented in Figures 6, 7, 8. The correlations were performed considering all the gestational weeks used in this study.

**TABLE 2:** Correlation matrix between the immunostainings

	IHH	PTCH1	SMO
IHH	–		
PTCH1	0.70	–	
SMO	0.70	0.30	–

*Note*: Very high correlation: 0.90–1.00. High correlation: 0.7–0.89. Moderate correlation: 0.5–0.69. Low correlation: 0.3–0.49. Insignificant correlation: 0–0.29.

## 4 DISCUSSION

In 1952, Symons pioneered the study of the development of the temporomandibular joint in human fetuses, laying the groundwork for subsequent research (Symons, 1952). Since then, a significant amount of new research on the subject has been published (Moffatt, 1957; Morimoto et al., 1987; Perry et al., 1985; Van der Linden et al., 1987).

It has been established that, in human specimens, chondrification of the mandibular condyle begins in the 9th week of gestation (Lee et al., 2001; Mérida‐Velasco et al., 1999; Mérida‐Velasco et al., 1999; Lee et al., 2001). In this study, we analyzed fetuses at fetal stages from 30 to 80 mm greatest length, a critical period for the formation of TMJ structures, as during this time, the mandibular condyle undergoes chondrification, and the superior and inferior joint cavities develop (cavitation period) (Mérida‐Velasco et al., 1999). This study is the first to evaluate the immunostaining of IHH, PTCH1, and SMO proteins in the TMJ of human fetuses during the third month of gestation.

In recent years, the development of the TMJ has been the subject of several investigations, mainly in animal models, focusing on the molecular signaling that regulates cellular differentiation and the growth of joint structures. The IHH pathway, in particular, has been extensively studied in rodents and other experimental models, demonstrating its critical role in regulating condylar growth (Bechtold et al., 2019; Koyama et al., 2007; Shibukawa et al., 2007; Yang et al., 2016).

The literature suggests that IHH signaling is crucial for the proliferation and differentiation of chondrocytes, having a direct impact on the formation of the condyle and the articular disc (Sun et al., 2024). On the other hand, the influence of movement on the joint cavitation process has been demonstrated (Murray & Drachm, 1969) and it has been shown that immobilization leads to the absence of joint cavities and skeletal abnormalities (Sperber et al., 2018). Similarly, joint movement stimulates condylar chondrification (Perry et al., 1985; Sperber et al., 2018). The initial reflex movements of the limbs and head in human embryos begin in the 6th week of development, as observed in real‐time ultrasound studies (de Vries et al., 1982; de Vries & Fong, 2006). Humphrey (1968) reported reflex mouth‐opening movements at 7–8 weeks in human specimens. These early oral movements have been suggested to occur at the joint between the incus and the posterior end of Meckel's cartilage, through the so‐called Meckelian articular complex (Humphrey, 1968; Merida‐Velasco et al., 1990). Recent ultrasonographic studies have provided a timeline of embryonic and fetal mobility, indicating that from the 19th week of gestation, the fetus can open its mouth and exhibit sucking‐swallowing movements, which begin around week 12 (Cabanyes Truffino, 2014). The formation of the joint cavities occurs between weeks 9 and 11 of development (Mérida‐Velasco et al., 1999), marking the onset of primary joint movements in the TMJ. These movements not only result from the structural differentiation of the mandibular condyle but also play a crucial role in regulating the molecular pathways responsible for TMJ development (Mérida‐Velasco et al., 2009). From the 12th week of gestation, once the joint cavities are fully formed, the synovial membrane becomes functional (Moraes et al., 2015). During this stage, IHH, PTCH1, and SMO‐mediated signaling undergo significant variations.

Our histological analysis also revealed a temporal association between the stage of Meckel's cartilage and the maturation of the mandibular condyle. At the 30 and 40 mm GL stages, Meckel's cartilage was clearly visible and corresponded with the early differentiation of the condylar layers, such as the chondroblastic and chondrocyte layers. As development progressed toward 60, 70, and 80 mm GL, we observed a gradual regression of Meckel's cartilage. In parallel, there was notable maturation of the condylar cartilage, including the appearance of the hypertrophic chondrocyte layer and the organization of the joint cavities. These findings suggest a coordinated timeline in which the involution of Meckel's cartilage parallels the structural and functional maturation of the TMJ, corroborating previous findings described in the literature (Merida‐Velasco et al., 1990; Mérida‐Velasco et al., 1999).

Our results show high IHH staining at the fetal Stage 30 mm GL, with its peak expression occurring at the beginning of this stage, coinciding with the onset of inferior joint cavity formation. A progressive decrease was observed until the stage of 60 mm GL. By the end of the stage of 60 mm, our findings revealed the appearance of a hypertrophic chondrocyte layer in the condyle, coinciding with the initiation of superior joint cavity formation. At the stage of 70 mm GL, IHH staining increased to 3.70%, and at this stage, the synovial membrane was clearly evident. During the stage of 80 mm GL, a decrease in IHH staining was observed, corresponding to the onset of the TMJ maturation period. These findings suggest that IHH plays an active role in early TMJ morphogenesis, not only in rats but also in humans.

For PTCH1, the peak immunostaining occurred at the stage of 30 mm GL, followed by a progressive decrease until the stage of 80 mm GL. The literature suggests that PTCH1 regulates IHH signaling by acting as its receptor and modulating the pathway's activity (Purcell et al., 2009).

SMO exhibited staining patterns similar to IHH, with its highest levels observed in the early weeks of condylar development and again at the stage of 80 mm GL, corresponding to the stage of cellular maturation (Mérida‐Velasco et al., 1999). This finding may suggest that SMO continues to play its role as a mediator of the IHH pathway, which is essential for TMJ morphogenesis, as SMO is a key protein in the IHH pathway, regulating cartilage cell proliferation and differentiation during embryonic development (Riobo et al., 2006; Ruiz i Altaba, 2011).

The results obtained corroborate with previous studies, such as those conducted by Shibukawa (Shibukawa et al., 2007) and Purcell (Purcell et al., 2009), which investigated the expression of IHH, PTCH1, and SMO in animal models, specifically in rats. These studies demonstrated the presence of IHH in the mandibular condyle during the embryonic period and provide important context for the present study, which investigates these proteins in human fetuses for the first time (Purcell et al., 2009; Shibukawa et al., 2007). Although a direct comparison is not possible, the findings from the existing literature help contextualize the role of IHH, PTCH1, and SMO in different developmental contexts of the TMJ.

The morphological findings and the immunohistochemical patterns observed in our study appear to be closely related and complementary. At early stages (30 and 40 mm GL), when the condylar cartilage is in its initial organization phase, we observed higher expression of IHH, PTCH1, and SMO, particularly in regions corresponding to the chondroblastic and chondrocyte layers. These proteins are known to regulate chondrocyte proliferation and early differentiation, which is consistent with the histological characteristics seen at these stages (Mau et al., 2007; Shibukawa et al., 2007). As development progresses (60–80 mm GL), and features such as the hypertrophic layer, joint cavities, and synovial membrane become more defined, we noted changes in expression patterns—especially a secondary peak of IHH and SMO—which may reflect their roles in condylar maturation and joint cavitation. This parallel between tissue architecture and protein dynamics reinforces the involvement of the Hedgehog pathway in distinct, temporally regulated phases of TMJ morphogenesis.

The analysis performed through software quantification revealed consistent expression patterns for the proteins IHH, PTCH1, and SMO across the stages of 30–80 mm GL. The trends of increase and decrease over time were clearly identifiable, suggesting a dynamic regulation of these proteins during mandibular condyle development. By objectively measuring the stained area and intensity, the software analysis minimized subjective interpretation.

IHH showed a marked increase in staining intensity during the stage of 70 mm, followed by a decrease in the last one, 80 mm, reflecting critical stages in tissue differentiation and joint maturation occurring near the 12th week gestational age. Similar temporal variations were observed for PTCH1 and SMO, each following patterns that align with their known biological roles in cartilage development and the regulation of the Hedgehog signaling pathway.

Although the small sample size limits the statistical power of the present study, the correlation analysis was used with an exploratory and descriptive purpose. The Spearman test allowed the identification of potential relationships between the protein expression patterns across the fetal stages analyzed. It is important to emphasize that these correlations are not intended to represent definitive statistical associations but rather to illustrate observable tendencies that may inform future studies with larger sample sizes. Given the biological plausibility of the results and their consistency with the morphological observations, the findings were included as preliminary data to support future investigations into the role of the IHH pathway in TMJ morphogenesis.

Altogether, the quantitative approach provided by software analysis reinforced the robustness of our findings, offering a detailed overview of proteins immunostaining during the critical phases of TMJ formation. In summary, our study suggests that the variation in the immunostaining of IHH, PTCH1, and SMO proteins in the mandibular condyle of human specimens during the third month of gestation may be related to joint mobility (Shea et al., 2015) and the development and growth of condylar cartilage during this critical period of TMJ formation.

In addition to advancing the understanding of the molecular regulation of human temporomandibular joint morphogenesis, our findings have both biological relevance and broader clinical and translational implications. The temporal variations observed in the expression of IHH, PTCH1, and SMO suggest that these proteins may serve as early molecular markers for the detection and diagnosis of craniofacial developmental anomalies. Given their central role in Hedgehog signaling, alterations in their activity could also represent future targets for precision therapies and gene therapy approaches. Moreover, insights into their expression dynamics may support genetic counseling in families affected by syndromes involving Hedgehog pathway mutations. Future studies should expand to larger human series, integrate molecular data with advanced prenatal imaging, and explore translational applications that may enable early intervention and novel therapeutic strategies for temporomandibular joint‐related disorders.

## AUTHOR CONTRIBUTIONS

**Filipe Santos da Silva:** Conceptualization; investigation; writing – original draft; methodology; writing – review and editing; software; formal analysis. **Carolina de Oliveira Gigek:** Methodology. **Andreia Fabiana Do Vale Franco:** Methodology. **Amanda Alves Ribeiro Massoni:** Methodology; software; formal analysis. **José Ramon Mérida‐Velasco:** Writing – review and editing. **Luís Otávio Carvalho de Moraes:** Supervision; resources; project administration; conceptualization; methodology; writing – review and editing.

## CONFLICT OF INTEREST STATEMENT

The authors declare no conflicts of interest.

## References

[ar70059-bib-0001] Alomar, X. , Medrano, J. , Cabratosa, J. , Clavero, J. A. , Lorente, M. , Serra, I. , Monill, J. M. , & Salvador, A. (2007). Anatomy of the temporomandibular joint. Seminars in Ultrasound, CT and MRI, 28(3), 170–183. 10.1053/j.sult.2007.02.002 17571700

[ar70059-bib-0002] Bechtold, T. E. , Kurio, N. , Nah, H.‐D. , Saunders, C. , Billings, P. C. , & Koyama, E. (2019). The roles of Indian Hedgehog signaling in TMJ formation. International Journal of Molecular Sciences, 20(24), 6300. 10.3390/ijms20246300 31847127 PMC6941023

[ar70059-bib-0003] Burch, J. G. (1970). Activity of the accessory ligaments of the temporomandibular joint. The Journal of Prosthetic Dentistry, 24(6), 621–628. 10.1016/0022-3913(70)90098-3 5273663

[ar70059-bib-0004] Cabanyes Truffino, J. (2014). El comportamiento fetal: una ventana al neurodesarrollo y al diagnóstico temprano. Pediatría Atención Primaria, 16(63), e101–e110. 10.4321/S1139-76322014000400012

[ar70059-bib-0005] Chen, K. , Quan, H. , Chen, G. , & Xiao, D. (2017). Spatio‐temporal expression patterns of Wnt signaling pathway during the development of temporomandibular condylar cartilage. Gene Expression Patterns, 25, 149–158. 10.1016/j.gep.2017.08.001 28800889

[ar70059-bib-0006] de Ferraris, G. , & Muñoz, A. C. (1999). Histologia y Embriologia Bucodental. Editorial Panamericana.

[ar70059-bib-0007] de Vries, J. I. P. , & Fong, B. F. (2006). Normal fetal motility: An overview. Ultrasound in Obstetrics & Gynecology, 27(6), 701–711. 10.1002/uog.2740 16710877

[ar70059-bib-0008] de Vries, J. I. P. , Visser, G. H. A. , & Prechtl, H. F. R. (1982). The emergence of fetal behaviour. I. Qualitative aspects. Early Human Development, 7(4), 301–322. 10.1016/0378-3782(82)90033-0 7169027

[ar70059-bib-0009] Herrera‐Valencia, A. , Ruiz‐Muñoz, M. , Martin‐Martin, J. , Cuesta‐Vargas, A. , & González‐Sánchez, M. (2020). Efficacy of manual therapy in temporomandibular joint disorders and its medium‐ and long‐term effects on pain and maximum mouth opening: A systematic review and meta‐analysis. Journal of Clinical Medicine, 9(11), 3404. 10.3390/jcm9113404 33114236 PMC7690916

[ar70059-bib-0010] Hinkle, D. E. , Wiersma, W. , & Jurs, S. G. (2003). Applied statistics for the behavioral sciences (Houghton Mifflin, 5a).

[ar70059-bib-0011] Humphrey, T. (1968). The development of mouth opening and related reflexes involving the oral area of human fetuses. Alabama Journal of Medical Sciences, 5(2), 126–157. 5675826

[ar70059-bib-0012] Idáñez‐Robles, A. M. , Obrero‐Gaitán, E. , Lomas‐Vega, R. , Osuna‐Pérez, M. C. , Cortés‐Pérez, I. , & Zagalaz‐Anula, N. (2023). Exercise therapy improves pain and mouth opening in temporomandibular disorders: A systematic review with meta‐analysis. Clinical Rehabilitation, 37(4), 443–461. 10.1177/02692155221133523 36263523

[ar70059-bib-0013] Koyama, E. , Ochiai, T. , Rountree, R. B. , Kingsley, D. M. , Enomoto‐Iwamoto, M. , Iwamoto, M. , & Pacifici, M. (2007). Synovial joint formation during mouse limb skeletogenesis: Roles of Indian Hedgehog signaling. Annals of the new York Academy of Sciences, 1116(1), 100–112. 10.1196/annals.1402.063 18083924 PMC2673545

[ar70059-bib-0014] Landini, G. , Martinelli, G. , & Piccinini, F. (2021). Colour deconvolution: Stain unmixing in histological imaging. Bioinformatics, 37(10), 1485–1487. 10.1093/bioinformatics/btaa847 32997742

[ar70059-bib-0015] Lee, S. K. , Kim, Y. S. , Oh, H. S. , Yang, K. H. , Kim, E. C. , & Chi, J. G. (2001). Prenatal development of the human mandible. Anatomical Record, 263(3), 314–325. 10.1002/ar.1110 11455541

[ar70059-bib-0016] Lu, K. , Ma, F. , Yi, D. , Yu, H. , Tong, L. , & Chen, D. (2022). Molecular signaling in temporomandibular joint osteoarthritis. Journal of Orthopaedic Translation, 32, 21–27. 10.1016/j.jot.2021.07.001 35591935 PMC9072795

[ar70059-bib-0017] Mau, E. , Whetstone, H. , Yu, C. , Hopyan, S. , Wunder, J. S. , & Alman, B. A. (2007). PTHrP regulates growth plate chondrocyte differentiation and proliferation in a Gli3 dependent manner utilizing hedgehog ligand dependent and independent mechanisms. Developmental Biology, 305(1), 28–39. 10.1016/j.ydbio.2007.01.031 17328886

[ar70059-bib-0018] Merida‐Velasco, J. R. , Rodríguez Vázquez, J. , & Jiménez Collado, J. (1990). Meckelial articular complex. Eur Arco Biology, 101, 447–453.

[ar70059-bib-0019] Mérida‐Velasco, J. R. , Rodríguez Vázquez, J. F. , De la Cuadra Blanco, C. , Campos López, R. , Sánchez, M. , & Mérida Velasco, J. A. (2009). Development of the mandibular condylar cartilage in human specimens of 10–15 weeks' gestation. Journal of Anatomy, 214(1), 56–64. 10.1111/j.1469-7580.2008.01009.x 19166473 PMC2667917

[ar70059-bib-0020] Mérida‐Velasco, J. R. , Rodríguez‐Vázquez, J. F. , Mérida‐Velasco, J. A. , Sánchez‐Montesinos, I. , Espín‐Ferra, J. , & Jiménez‐Collado, J. (1999). Development of the human temporomandibular joint. Anatomical Record, 255(1), 20–33. 10.1002/(SICI)1097-0185(19990501)255:1<20::AID-AR4>3.0.CO;2-N 10321990

[ar70059-bib-0021] Moffatt, B. (1957). The prenatal development of the human temporomandibular joint. In Contributions to embryology (Vol. 36, pp. 21–28). Carnegie Institution.

[ar70059-bib-0022] Moraes, L. O. C. , Lodi, F. R. , Gomes, T. S. , Marques, S. R. , Fernandes Junior, J. A. , Oshima, C. T. F. , & Alonso, L. G. (2008). Immunohistochemical expression of collagen type IV antibody in the articular disc of the temporomandibular joint of human fetuses. Italian Journal of Anatomy and Embryology, 113(2), 91–95. 18702236

[ar70059-bib-0023] Moraes, L. O. C. , Tedesco, R. C. , Arraez‐Aybar, L. A. , Klein, O. , Merida‐Velasco, J. R. , & Alonso, L. G. (2015). Development of synovial membrane in the temporomandibular joint of the human fetus. European Journal of Histochemistry, 59(4), 2569. 10.4081/ejh.2015.2569 26708184 PMC4698616

[ar70059-bib-0024] Morimoto, K. , Hashimoto, N. , & Suetsugu, T. (1987). Prenatal developmental process of human temporomandibular joint. Journal of Prosthetic Dentistry, 57(6), 723–730. 10.1016/0022-3913(87)90372-6 3473232

[ar70059-bib-0025] Murray, P. D. F. , & Drachm, D. B. (1969). The role of movement in the development of joints and related structures: The head and neck in the chick embryo. Development, 22(3), 349–371. 10.1242/dev.22.3.349 5360022

[ar70059-bib-0026] Nickel, J. C. , Iwasaki, L. R. , Gonzalez, Y. M. , Gallo, L. M. , & Yao, H. (2018). Mechanobehavior and ontogenesis of the temporomandibular joint. Journal of Dental Research, 97(11), 1185–1192. 10.1177/0022034518786469 30004817 PMC6151909

[ar70059-bib-0027] Niu, Q. , Li, F. , Zhang, L. , Xu, X. , Liu, Y. , Gao, J. , & Feng, X. (2016). Role of the Wnt/β‐catenin signaling pathway in the response of chondrocytes to mechanical loading. International Journal of Molecular Medicine, 37(3), 755–762. 10.3892/ijmm.2016.2463 26821383

[ar70059-bib-0028] O'Rahilly, R. , & Müller, F. (2001). Human embryology & teratology (3rd ed.). Wiley‐Liss.

[ar70059-bib-0029] O'Rahilly, R. , & Müller, F. (2010). Developmental stages in human embryos: Revised and new measurements. Cells, Tissues, Organs, 192(2), 73–84. 10.1159/000289817 20185898

[ar70059-bib-0030] Pacifici, M. (2006). Cellular and molecular mechanisms of synovial joint and articular cartilage formation. Annals of the new York Academy of Sciences, 1068(1), 74–86. 10.1196/annals.1346.010 16831907 PMC2697570

[ar70059-bib-0031] Perry, H. , Xu, Y. , & Forbes, D. (1985). The embryology of the temporomandibular joint, 3, 125–132. 10.1080/08869634.1985.11678094 3855934

[ar70059-bib-0032] Purcell, P. , Joo, B. W. , Hu, J. K. , Tran, P. V. , Calicchio, M. L. , O'Connell, D. J. , Maas, R. L. , & Tabin, C. J. (2009). Temporomandibular joint formation requires two distinct hedgehog‐dependent steps. Proceedings of the National Academy of Sciences of the United States of America, 106(43), 18297–18302. 10.1073/pnas.0908836106 19815519 PMC2775291

[ar70059-bib-0033] Riobo, N. A. , Lu, K. , & Emerson, J. C. P. (2006). Hedgehog signal transduction: Signal integration and cross talk in development and cancer. Cell Cycle, 5(15), 1612–1615. 10.4161/cc.5.15.3130 16880744

[ar70059-bib-0034] Roberts, W. E. , & Stocum, D. L. (2018). Part II: Temporomandibular joint (TMJ)—Regeneration, degeneration, and adaptation. Current Osteoporosis Reports, 16(4), 369–379. 10.1007/s11914-018-0462-8 29943316

[ar70059-bib-0035] Ruiz i Altaba, A. (2011). Hedgehog signaling and the Gli code in stem cells, cancer, and metastases. Science Signaling, 4(200), 9. 10.1126/scisignal.2002540 22114144

[ar70059-bib-0036] Shea, C. A. , Rolfe, R. A. , & Murphy, P. (2015). The importance of foetal movement for co‐ordinated cartilage and bone development in utero: Clinical consequences and potential for therapy. Bone & Joint Research, 4(7), 105–116. 10.1302/2046-3758.47.2000387 26142413 PMC4602203

[ar70059-bib-0037] Shibukawa, Y. , Young, B. , Wu, C. , Yamada, S. , Long, F. , Pacifici, M. , & Koyama, E. (2007). Temporomandibular joint formation and condyle growth require Indian hedgehog signaling. Developmental Dynamics, 236(2), 426–434. 10.1002/dvdy.21036 17191253

[ar70059-bib-0038] Sperber, G. H. , Sperber, S. M. , & Guttmann, G. D. (2018). Craniofacial embryogenetics and development (3rd ed.). PMPH‐USA, Ltd.

[ar70059-bib-0039] St‐Jacques, B. , Hammerschmidt, M. , & McMahon, A. P. (1999). Indian hedgehog signaling regulates proliferation and differentiation of chondrocytes and is essential for bone formation. Genes & Development, 13(16), 2072–2086. 10.1101/gad.13.16.2072 10465785 PMC316949

[ar70059-bib-0040] Stocum, D. L. , & Roberts, W. E. (2018). Part I: Development and physiology of the temporomandibular joint. Current Osteoporosis Reports, 16(4), 360–368. 10.1007/s11914-018-0447-7 29948821

[ar70059-bib-0041] Sun, Q. , Huang, J. , Tian, J. , Lv, C. , Li, Y. , Yu, S. , Liu, J. , & Zhang, J. (2024). Key roles of Gli1 and Ihh signaling in craniofacial development. Stem Cells and Development, 33(11–12), 251–261. 10.1089/scd.2024.0036 38623785

[ar70059-bib-0042] Symons, N. B. B. (1952). The development of the human mandibular joint. Journal of Anatomy, 86(3), 326–332. 12980883 PMC1273756

[ar70059-bib-0043] Takagi, K. , Takada, T. , & Amano, H. (2005). A high peripheral microvessel density count correlates with a poor prognosis in pancreatic cancer. Journal of Gastroenterology, 40(4), 402–408. 10.1007/s00535-004-1556-x 15870976

[ar70059-bib-0044] Takagi, R. , Ohashi, Y. , Yoshida, S. , & Kobayashi, S. (1989). Microvascular architecture of the temporomandibular joint in the adult rabbit as studied by the injection replica SEM method. 1. Rest position of jaws. Nihon Ago Kansetsu Gakkai Zasshi, 1(1), 102–109. 2489178

[ar70059-bib-0045] Van der Linden, E. J. , Burdi, A. R. , & de Jongh, H. J. (1987). Critical periods in the prenatal morphogenesis of the human lateral pterygoid muscle, the mandibular condyle, the articular disk, and medial articular capsule. American Journal of Orthodontics and Dentofacial Orthopedics, 91(1), 22–28. 10.1016/0889-5406(87)90205-8 3467577

[ar70059-bib-0046] Yang, L. , Gu, S. , Ye, W. , Song, Y. , & Chen, Y. (2016). Augmented Indian hedgehog signaling in cranial neural crest cells leads to craniofacial abnormalities and dysplastic temporomandibular joint in mice. Cell and Tissue Research, 364(1), 105–115. 10.1007/s00441-015-2306-5 26553654 PMC4930651

